# Genome-wide analyses of the bZIP family reveal their involvement in the development, ripening and abiotic stress response in banana

**DOI:** 10.1038/srep30203

**Published:** 2016-07-22

**Authors:** Wei Hu, Lianzhe Wang, Weiwei Tie, Yan Yan, Zehong Ding, Juhua Liu, Meiying Li, Ming Peng, Biyu Xu, Zhiqiang Jin

**Affiliations:** 1 Key Laboratory of Biology and Genetic Resources of Tropical Crops, Institute of Tropical Bioscience and Biotechnology, Chinese Academy of Tropical Agricultural Sciences, Haikou, Hainan, 571101, China; 2 School of Life Science and Engineering, Henan University of Urban Construction, Pingdingshan, Henan, 467044, China; 3 Key Laboratory of Genetic Improvement of Bananas, Hainan province, Haikou Experimental Station, China Academy of Tropical Agricultural Sciences, Haikou, Hainan, 570102, China

## Abstract

The leucine zipper (bZIP) transcription factors play important roles in multiple biological processes. However, less information is available regarding the bZIP family in the important fruit crop banana. In this study, 121 bZIP transcription factor genes were identified in the banana genome. Phylogenetic analysis showed that MabZIPs were classified into 11 subfamilies. The majority of *MabZIP* genes in the same subfamily shared similar gene structures and conserved motifs. The comprehensive transcriptome analysis of two banana genotypes revealed the differential expression patterns of *MabZIP* genes in different organs, in various stages of fruit development and ripening, and in responses to abiotic stresses, including drought, cold, and salt. Interaction networks and co-expression assays showed that group A MabZIP-mediated networks participated in various stress signaling, which was strongly activated in *Musa* ABB Pisang Awak. This study provided new insights into the complicated transcriptional control of *MabZIP* genes and provided robust tissue-specific, development-dependent, and abiotic stress-responsive candidate *MabZIP* genes for potential applications in the genetic improvement of banana cultivars.

In plants, transcription factors (TFs), which function in regulating gene expression by interacting with the promoter regions of downstream genes, play crucial roles in multiple plant biological processes. TFs can be grouped into more than 60 families based on their structural similarities of conserved domains^1^. Among them, the leucine zipper (bZIP) transcription factors, one of the largest gene families, play vital roles involved in plant development and responses to various stresses^2^,^3^. The bZIP proteins contain a conserved bZIP domain that is composed of two functional regions of a highly conserved basic region and a less conserved leucine zipper motif^4^. The basic region contains an invariant N-x7-R/K motif within 16 amino acid residues that allow for nuclear localization and DNA-binding. The leucine zipper motif, which consists of several repeats of leucine or other hydrophobic amino acids, is responsible for specific recognition and dimerization^4^.

To date, based on genome sequencing, the *bZIP* gene family has been identified in several plant genomes. It has been reported that there are 75 *bZIP* genes in *Arabidopsis* (*Arabidopsis thaliana* L.)^3^, 89 in rice (*Oryza sativa* L.)^5^, 55 in grapevine (*Vitis vinifera* L.)^6^, 64 in cucumber (*Cucumis sativus* L.)^7^, 92 in sorghum (*Sorghum bicolor* L.)^8^, 96 in *B. distachyon* (*Brachypodium distachyon* L.)^9^ and 125 in maize (*Zea mays* L.)^10^. Biochemical and functional analyses have demonstrated that the bZIP family is involved in many essential plant biological processes, including the plant developmental processes of organs differentiation, floral induction, vascular development, embryogenesis and seed maturation^11^,^12^,^13^. For example, *AtbZIP34* functions in *Arabidopsis* pollen wall patterning through regulating several metabolic pathways of lipid metabolism and/or cellular transport^14^. A sugar-regulated gene, *AtbZIP1*, participates in sugar signaling and affects early seedling growth and development^15^. In addition, the bZIP TFs have also been shown to act as key components in the abiotic and biotic stresses signaling pathways, including drought^16^,^17^, high salinity^18^, heat^19^, cold^17^,^20^, abscisic acid (ABA)^21^,^22^ and some pathogen infections^23^,^24^. In particular, the group A *bZIP* genes, which are named as ABA-responsive element binding proteins (AREB) or ABRE binding factors (ABF), have been functionally characterized as important players in ABA or stress signaling^21^,^22^. The group B bZIP members have transmembrane domain and specific domain at C-terminus which are important for ER stress response, and the group S bZIPs are also induced under environmental stress^3^. Furthermore, bZIP mediating the crosstalk network of stress and development has also been revealed. C/S1 group bZIP transcription factors have been shown to be involved in the network of stress response and development by expression profile analysis^25^,^26^. Together, these studies have revealed the important roles of the *bZIP* gene family in regulating plant development and responses to multiple stresses.

Banana (*Musa acuminata* L.) is a large monocotyledonous herbaceous plant that is widely distributed throughout tropical and subtropical countries. Banana is not only the most popular fruit but also one of the largest fruit crops, which is vital for food security for millions of people around the world^27^. Compared with some other crops, banana research has developed slowly, because banana is only planted as food for the largely impoverished continent of Africa^27^. In spite of the economic and social importance of banana and the critical role of bZIP transcription factors in the plant development and stress responses, only one *MabZIP* have been cloned and characterized^28^. Characterizing genes involved in the metabolic pathways and signal transduction pathways based on complete genome sequences is important to illuminate cellular biological processes^29^. The banana genome was completely sequenced in 2012 using the *Musa acuminata* doubled-haploid genotype (A genome)^30^, which provides an opportunity to further advance systematic analyses and for the functional characterization of *MabZIP* gene families.

In this study, we identified 121 *MabZIPs* from banana genome and investigated their phylogenetic relationship, protein motifs, gene structure, and expression patterns in diverse tissues, in different stages of fruit development and ripening, and responses to abiotic stress. Further, we characterized the interaction networks and co-expression patterns of group A MabZIPs in response to abiotic stress. This comprehensive study could increase our understanding of *MabZIP* associated with developmental processes and abiotic stress responses, and would establish a crucial foundation for future studies of crop improvement.

## Results

### Identification and phylogenetic analysis of banana *bZIPs*

To identify all bZIP family transcriptional factors in banana, Hidden Markov Model searches using the bZIP domains (PF00170) as queries, as well as BLAST searches using *Arabidopsis* and rice bZIP sequences as queries, were performed in the banana genome database. After validating the bZIP domain using the CDD and PFAM databases, a total of 121 MabZIP proteins were identified. The 121 predicted MabZIP proteins ranged from 121 (*MabZIP119*) to 616 (*MabZIP69*) amino acid residues with the relative molecular mass varying from 13.56 to 66.63 kDa, and the theoretical isoelectric points (PIs) are predicted to range from 5.27 (*MabZIP48*) to 10.68 (*MabZIP46*) (Supplementary Table S1).

**Figure 1:**
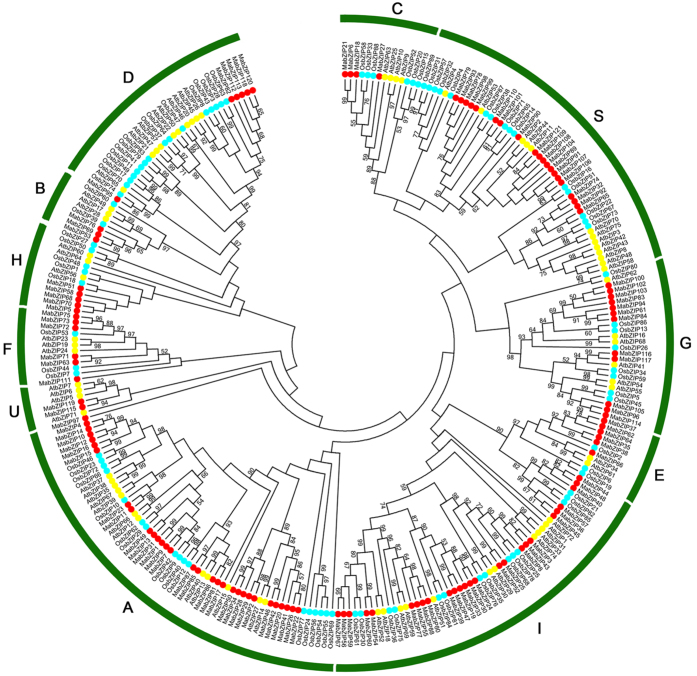
Phylogenetic analysis of the bZIPs from *Arabidopsis*, rice and banana. The Neighbor-joining (NJ) tree was drawn using MEGA 5.0 with 1000 bootstrap. 11 groups were shown as A to I, S and U.

To investigate the evolutionary relationship of bZIP family proteins, a neighbor-joining (NJ) tree was constructed with bZIP proteins from banana, rice and *Arabidopsis* (Fig. 1). Based on the phylogenetic tree, all the bZIPs proteins were grouped into 11 clusters, named as groups A to I, S and U, similar to those in *Arabidopsis* and grape^3^,^6^. Subfamilies A, I and S are large with more than 20 MabZIP members, whereas the other subfamilies each contain less than 12 MabZIP proteins, suggesting the bZIP family differentiated in banana with diverse functions. Some orthologous *bZIPs* between banana, rice and *Arabidopsis* have also been identified from evolutionary analysis, indicating that some ancestral *bZIP* existed prior to the divergence of banana, rice and *Arabidopsis*. Generally, bZIPs from banana have closer relationships with the bZIPs from rice than that from Arabidopsis, which is accord with the current understanding of plant evolutionary history.

### Conserved motifs and gene structure analyses of banana bZIPs

**Figure 2:**
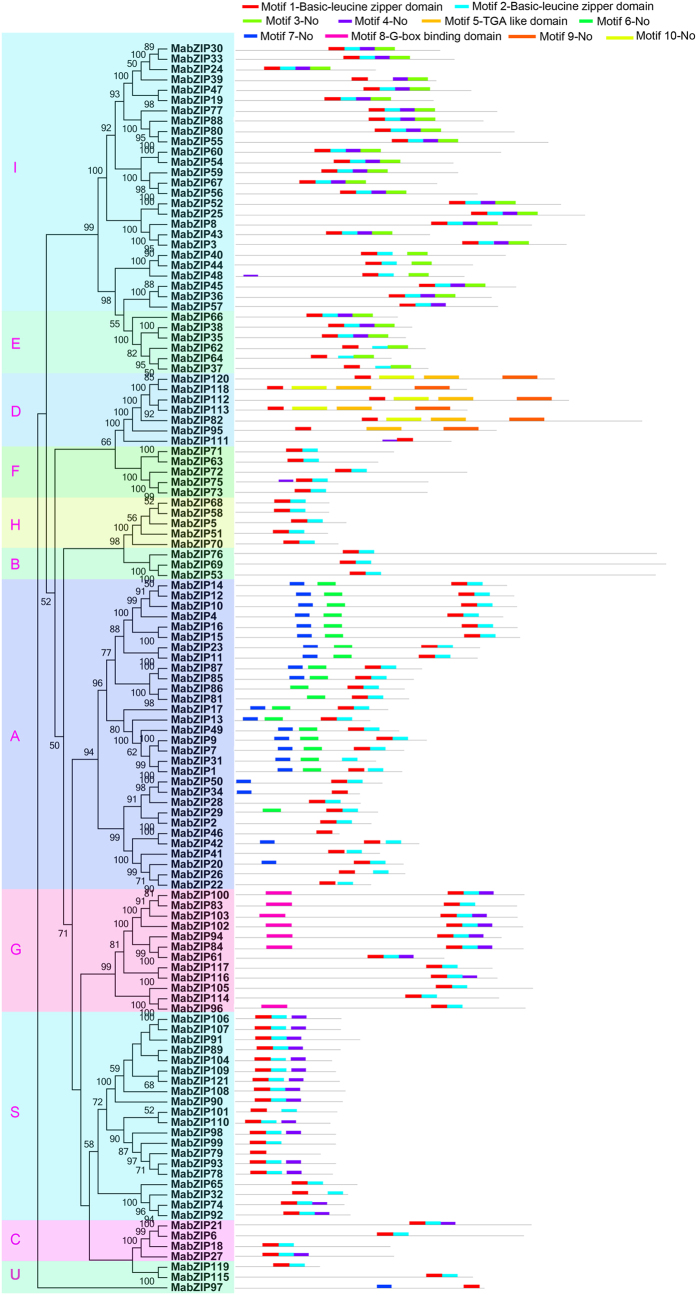
Phylogenetic and motif analyses of MabZIPs. All motifs were identified by MEME database with the complete amino acid sequences of MabZIPs. The classification of MabZIPs were shown as A to I, S and U based on the phylogenetic relationship.

To obtain insight into the divergence and function of the MabZIP proteins, a total of 10 conserved motifs were captured by MEME software and annotated with the InterPro database (Fig. 2). All the MabZIPs contain the basic leucine zipper domain of motif 1, and most of the MabZIPs, except for the D group, also contain the basic leucine zipper domain of motif 2. The D group contains the TGA-like domain of motif 5; and the G group contains the motif 8 which was annotated as a G-box binding domain. The motif analyses showed that most conserved motifs existed in the same group. Most of the bZIP members in group A contain motifs 6 and 7; most of the bZIPs in subfamilies I and E share motifs 3 and 4; group D shares the same motifs of 5, 9 and 10; and most of the S and C group proteins harbor motif 4. These results suggest that the MabZIPs clustered in the same group share similar amino acid sequences. In addition, the results can further support the phylogenetic analyses of banana bZIPs.

**Figure 3:**
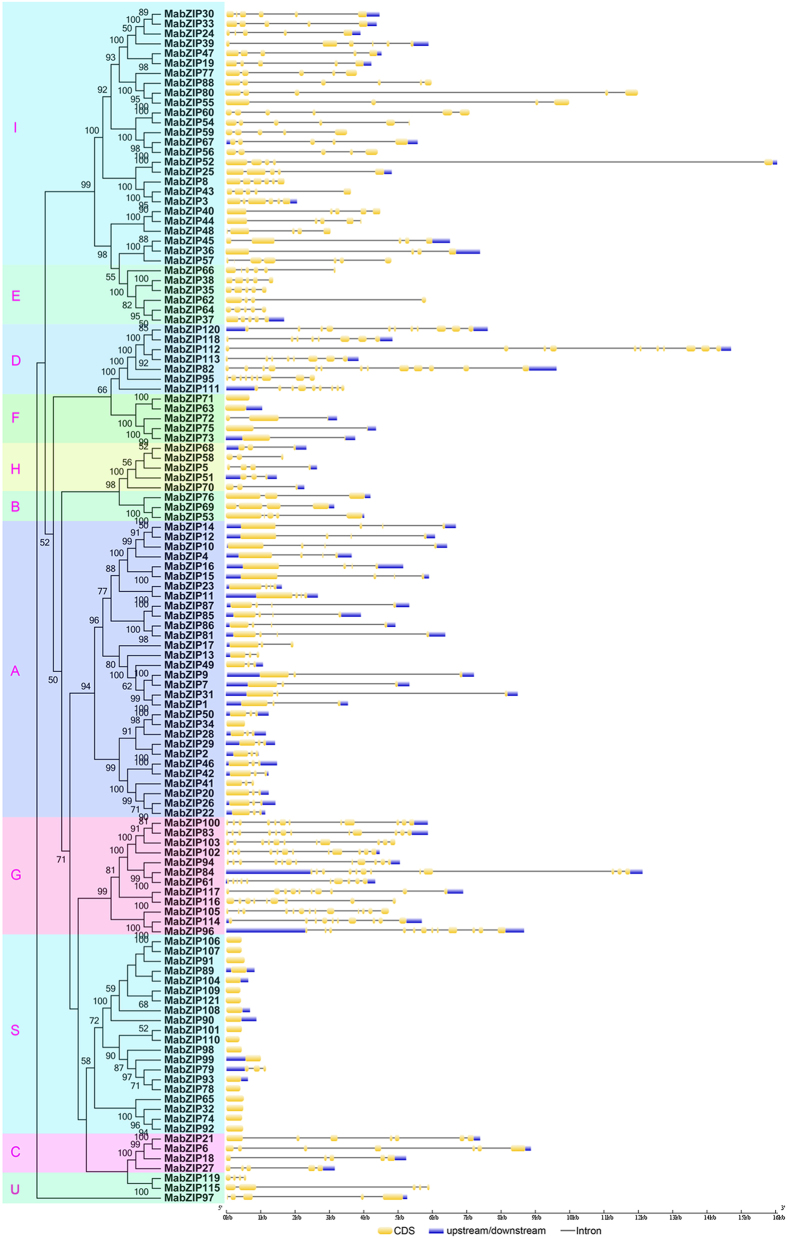
Gene structure analyses of *MabZIPs*. Exon-intron structure analyses were performed by GSDS database. The blue boxes indicate upstream/downstream, the yellow boxes indicate exons, and the black lines indicate introns.

Exon-intron organizations of the 121 *MabZIP* genes were also examined to better understand their structural evolution. As shown in Fig. 3, only 22 of a total of 121 *MabZIPs* have one exon, and the intronless genes were clustered in the S and F groups. The other *MabZIPs* contain exons with numbers varying from 2 to 13, and the genes with more than 9 exons were grouped in the D and G groups. This suggested that similar exon-intron organizations of *MabZIPs* exist in the same group and the gene structure might be meaningful for gene evolution.

### Expression profiles of *MabZIP* genes in different organs of two banana varieties

**Figure 4:**
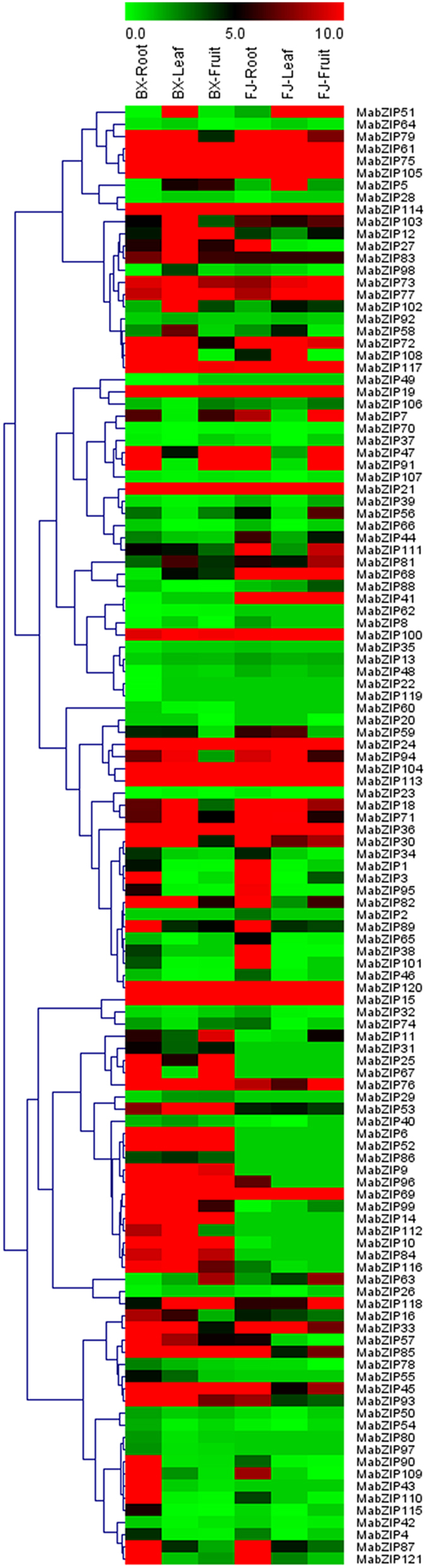
Expression patterns of *MabZIPs* in roots, leaves, and fruits of BX and FJ. The heat map with clustering was created based on the FPKM value of *MabZIPs*. Differences in gene expression changes are shown in color as the scale.

To investigate the organ expression patterns of *bZIP* genes in banana, roots, leaves, and fruits of BaXi Jiao (BX) and Fen Jiao (FJ) were sampled for RNA-seq assays. Among the 121 *MabZIP* genes, 120 genes (except for *MabZIP17*) showed expression in at least one tested organ of two varieties (Fig. 4; Supplementary Table S2).

For BX, 117 (97.5%), 106 (88.3%) and 103 (85.8%) *MabZIPs* were expressed in roots, leaves and fruits, respectively, among which 44 (36.7%), 46 (38.3%), and 29 (24.2%) genes showed high expression levels (value > 10) in roots, leaves, and fruits, respectively. Additionally, 17 (14.2%) genes had high transcriptional expression levels (value > 10) in all of the 3 tested organs.

For FJ, 98 (81.7%), 89 (74.2%) and 81 (67.5%) *MabZIP* genes were expressed in roots, leaves, and fruits, respectively, among which 37 (30.8%), 26 (21.7%) and 24 (20.0%) genes showed high expression levels (value > 10) in roots, leaves and fruits, respectively. Further, 17 (14.2%) genes had high expression levels (value > 10) in all tested organs.

By comparing the organ expression profiles of *MabZIPs* between BX and FJ, 119 genes of BX and 100 genes of FJ showed expression in all tested organs, indicating that there were more genes expressed in BX than in FJ. Generally, *MabZIPs* showed similar expression patterns in BX and FJ, with more *MabZIP* genes showing high expression in the roots and leaves than in the fruits. However, some gene expression patterns were different in the two banana varieties, e.g., 11 genes (*MabZIP-6*, *-9*, *-10*, *-14*, *-25*, *-43*, *-52*, *-67*, *-90*, *-99*, and *-110*) had high expression levels (value > 10) in BX roots, while they showed low expression levels (value < 5) in FJ roots. A total of 17 genes (*MabZIP-6*, *-9*, *-10*, *-12*, *-14*, *-27*, *-52*, *-53*, *-82*, *-84*, *-85*, *-93*, *-96*, *-99*, *-102*, *-112* and *-116*) showed high expression (value > 10) levels in BX leaves but low expression (value < 5) in FJ leaves. There were 9 genes (*MabZIP-6*, *-10*, *-12*, *-14*, *-25*, *-52*, *-53*, *-67* and *-96*) that showed high expression levels (value > 10) in BX fruits but low expression levels (value < 5) in FJ fruits. These results suggested that *MabZIP* genes had differential roles in the different organs of the two banana varieties. Additionally, 13 genes (*MabZIP-15*, *-19*, *-21*, *-24*, *-36*, *-61*, *-69*, *-75*, *-104*, *-105*, *-113*, *-114* and *-120*) showed high expression (value > 10) in all of the tested organs in both BX and FJ, indicating their important roles in organ development. Together, these organ expression profiles of the *MabZIP* genes in different varieties may provide insight for future studies on organ development and function.

### Expression profiles of *MabZIP* genes in different stages of fruit development and ripening of two banana varieties

**Figure 5:**
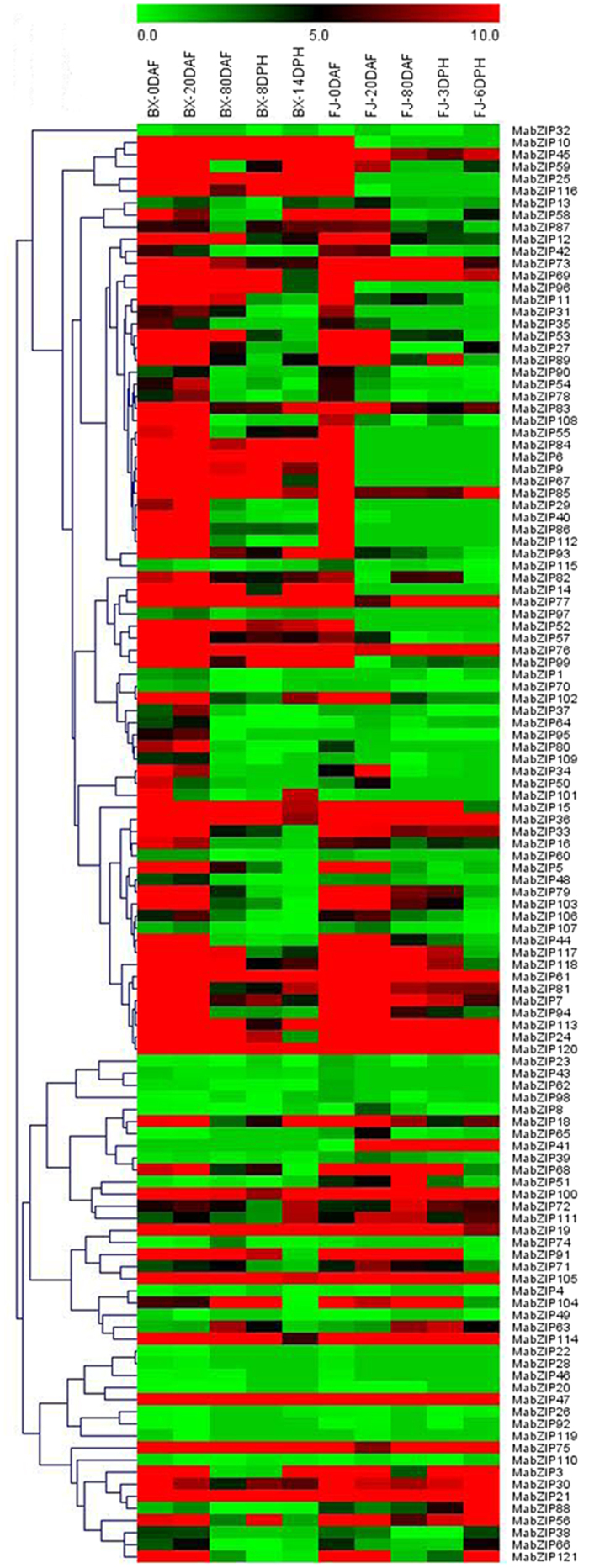
Expression patterns of *MabZIPs* in different stages of fruit development and ripening in BX and FJ varieties. The heat map with clustering was created based on the FPKM value of *MabZIPs*. Differences in gene expression changes are shown in color as the scale.

To obtain insight into the roles of the *MabZIP* genes in fruit development and ripening of banana, the expression patterns of the *MabZIP* genes were detected in fruits sampled from 0, 20, and 80 days after flowering (DAF) of the BX and FJ varieties, 8 and 14 days postharvest (DPH) of the fruits of BX, and 3 and 6 DPH of the fruits of FJ (Fig. 5; Supplementary Table S3). Among the 121 *MabZIPs*, 119 genes showed expression in different stages of fruit development and ripening.

For BX, 116 (97.5%), 117 (98.3%), 107 (89.9%), 101 (84.9%), 99 (83.2%) *MabZIPs* were expressed at 0 DAF, 20 DAF, 80 DAF, 8 DPH and 14 DPH, among which 61 (51.3%), 63 (52.9%), 29 (24.4%), 24 (20.2%) and 22 (18.5%) genes, respectively, showed high expression levels (value > 10) at each stage. A total of 11 genes (*MabZIP-6*, *-10*, *-19*, *-21*, *-25*, *-45*, *-47*, *-61*, *-75*, *-76* and *-77*) exhibited high transcript accumulation (value > 10) at all stages of fruit development and ripening.

For FJ, 118 (99.2%), 96 (80.7%), 81 (68.1%), 86 (72.3%) and 80 (67.2%) *MabZIPs* expressed at 0 DAF, 20 DAF, 80 DAF, 3 DPH and 6 DPH, among which 62 (52.1%), 39 (32.8%), 24 (20.2%), 22 (18.5%) and 19 (16.0%) genes, respectively, showed high expression levels (value > 10) at each stage. Additionally, 9 genes (*MabZIP-21*, *-24*, *-36*, *-47*, *-61*, *-100*, *-105*, *-114* and *-120*) showed high transcript accumulation (value > 10) at all stages of fruit development and ripening.

A comparison of the expression profiles of the *MabZIPs* at distinct stages of fruit development and ripening in BX and FJ indicated that 94 genes showed expression at all stages tested in BX, whereas 75 genes were expressed at all stages tested in FJ. Generally, the *MabZIP* expression profiles were similar in BX and FJ, with more highly expressed genes (value > 10) at 0 and 20 DAF compared with the other stages, indicating that the *MabZIPs* had important roles in the early fruit developmental stages of 0 and 20 DAF in both BX and FJ. Notably, more genes showed high expression levels in BX than in FJ. A total of 13 genes (*MabZIP-6*, *-9*, *-11*, *-29*, *-40*, *-55*, *-67*, *-84*, *-86*, *-93*, *-96*, *-108* and *-112*) showed high expression (value > 10) in BX but low expression (value < 5) in FJ at 20 DAF, and 11 genes (*MabZIP-6*, *-9*, *-10*, *-14*, *-25*, *-52*, *-67*, *-84*, *-93*, *-99* and *-116*) had higher expression levels (value > 10) at three subsequent stages in BX compared with FJ. These findings implied that the *MabZIP* genes had significant transcriptional responses during the fruit development and post-harvest ripening stages of BX. Additionally, *MabZIP21*, *MabZIP47* and *MabZIP61* showed high expression (value > 10) in all tested stages of BX and FJ, suggesting that these three genes might play extensive and vital roles during the banana developmental and ripening processes.

### Expression profiles of *MabZIP* genes under abiotic stress in two banana varieties

**Figure 6:**
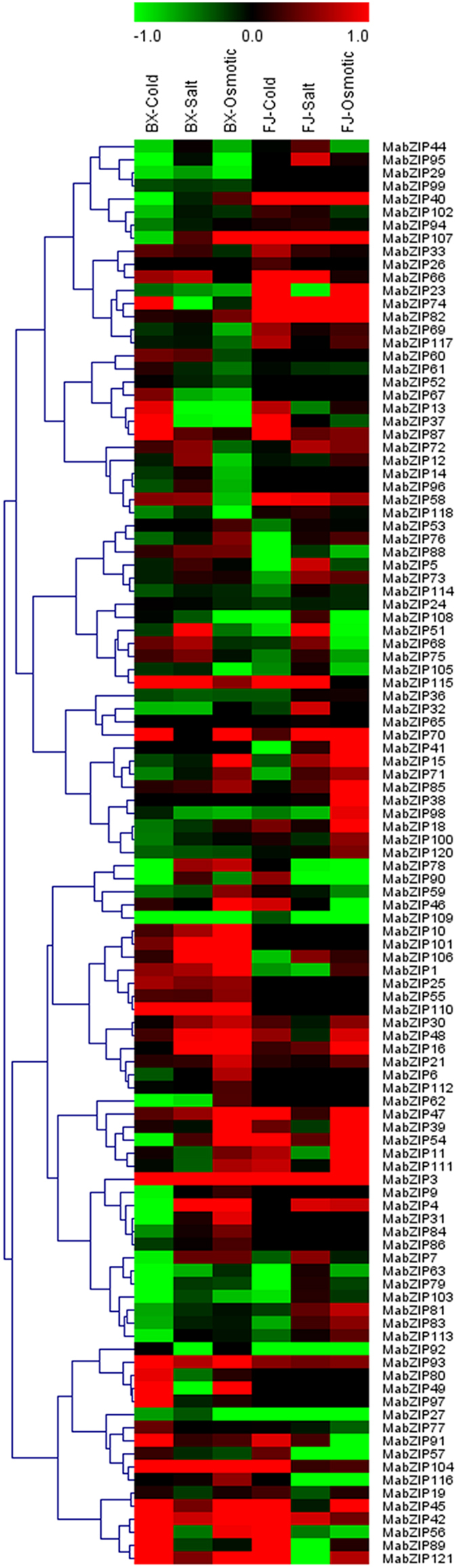
Expression patterns of *MabZIPs* in response to cold, salt, and osmotic treatments in BX and FJ varieties. Log2 based FPKM value was used to create the heat map. Differences in gene expression changes are shown in color as the scale.

To obtain insight into the expression profiles of *MabZIPs* in banana response to abiotic stress, the leaves of BX and FJ under cold, salt and osmotic treatments were sampled respectively for RNA-seq analysis (Fig. 6; Supplementary Table S4). A total of 109 genes of all of the 121 *MabZIPs* showed transcriptional changes after abiotic stress treatments in the BX and FJ varieties.

For BX, 50 (45.9%), 54 (49.5%) and 57 (52.3%) genes showed upregulation, while 54 (49.5%), 50 (45.9%) and 45 (41.3%) genes showed downregulation under cold, salt and osmotic stress, respectively. Further, 16, 8 and 21 *MabZIP* genes were significantly upregulated (value > 1) by cold, salt and osmotic stress, and 10, 5, and 6 genes were significantly downregulated (value < −1) under cold, salt and osmotic stress treatments, respectively. Additionally, *MabZIP104* and *MabZIP110* showed significant upregulation and *MabZIP109* showed significantly downregulation under all the three tested stresses.

For FJ, 45 (41.3%), 55 (50.5%) and 51 (46.8%) *MabZIP* genes showed upregulation, while 36 (33.0%), 29 (26.6%) and 32 (29.4%) genes showed downregulation under cold, salt and osmotic stress, respectively. Among them, 19, 9 and 19 *MabZIP* genes showed significant upregulation (value > 1) by cold, salt and osmotic stress, respectively, and 9, 8 and 10 genes were significantly downregulated (value < −1) in response to the respective abiotic stress treatments. Additionally, 5 genes (*MabZIP-3*, *-40*, *-74*, *-82* and *-107*) had significantly increased transcription and 2 genes (*MabZIP27* and *MabZIP92*) showed significantly reduced transcription in response to the three tested stresses.

From these results, it is clear that more genes were significantly upregulated (value > 1) in FJ than in BX under cold and salt treatments, Furthermore, the number of genes that were significantly upregulated and downregulated in response to the three tested abiotic stresses were higher in FJ than in BX.

### Validation of the differentially expressed bZIP genes by qRT-PCR analysis

**Figure 7:**
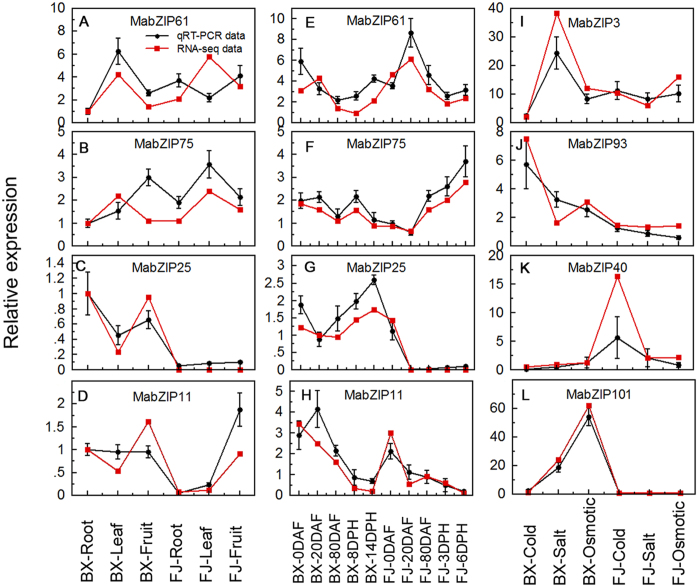
Relative expression of MabZIP genes in BX and FJ by qRT-PCR. (**A**–**D**) expression patterns of MabZIP61, MabZIP75, MabZIP25 and MabZIP11 in different organs. The mRNA fold difference was relative to that of BX-root samples used as calibrator. (**E**–**H**) expression patterns of MabZIP61, MabZIP75, MabZIP25 and MabZIP11 in different stages of fruit development and ripening. The mRNA fold difference was relative to that of BX-0DAF samples used as calibrator. (**I**–**L**) expression patterns of MabZIP3, MabZIP93, MabZIP40 and MabZIP101 in response to cold, salt and osmotic stresses. The mRNA fold difference was relative to that of untreated samples used as calibrator. Data are means ± SD of n = 3 biological replicates.

According to the RNA-seq data, MabZIP61 and MabZIP75 showed high expression levels (value > 7) in all three tested organs (root, leaf, and fruit) and all tested fruit development and ripening stages of BX and FJ. MabZIP25 and MabZIP11 showed different expression patterns between BX and FJ in the organs and fruit development and ripening stages. MabZIP3 and MabZIP93 were upregulated by cold, salt and osmotic stresses in BX and/or FJ. MabZIP40 and MabZIP101 had different expression patterns between BX and FJ under abiotic stress treatments. These eight differentially expressed MabZIP genes were selected for qRT-PCR analysis to validate the RNA-seq data. After normalization, we found that the majority of selected MabZIP genes, except for MabZIP61 in FJ leaf, MabZIP75 and MabZIP11 in BX fruit, and MabZIP61, MabZIP75 and MabZIP11 in BX 20 DAF, showed the same trend and consistent results between RNA-seq data and qRT-PCR data (Fig. 7). These results indicate that RNA-seq data are suitable for supplying the expression patterns of bZIP genes in two banana varieties.

### bZIP family interaction networks and their co-expression under abiotic stress

**Figure 8:**
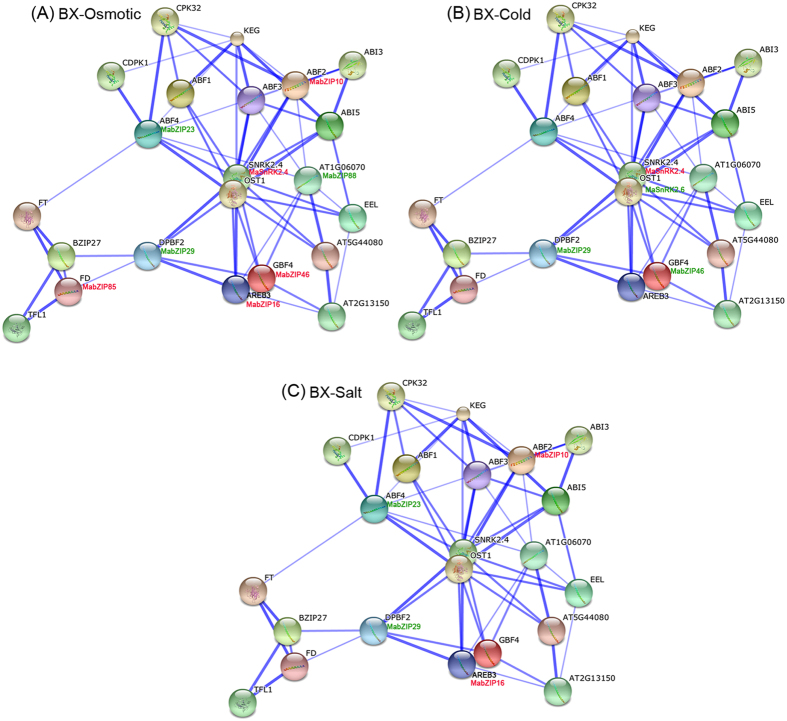
Interaction network and co-expression analyses of bZIPs under osmotic (**A**), cold (**B**), and salt (**C**) treatments in BX and related genes in Arabidopsis. Line thickness relates to combined score. The genes marked with red show upregulation (Log2 based FPKM value > 0.5) in BX. The genes marked with green show downregulation (Log2 based FPKM value < −0.5) in BX.

**Figure 9:**
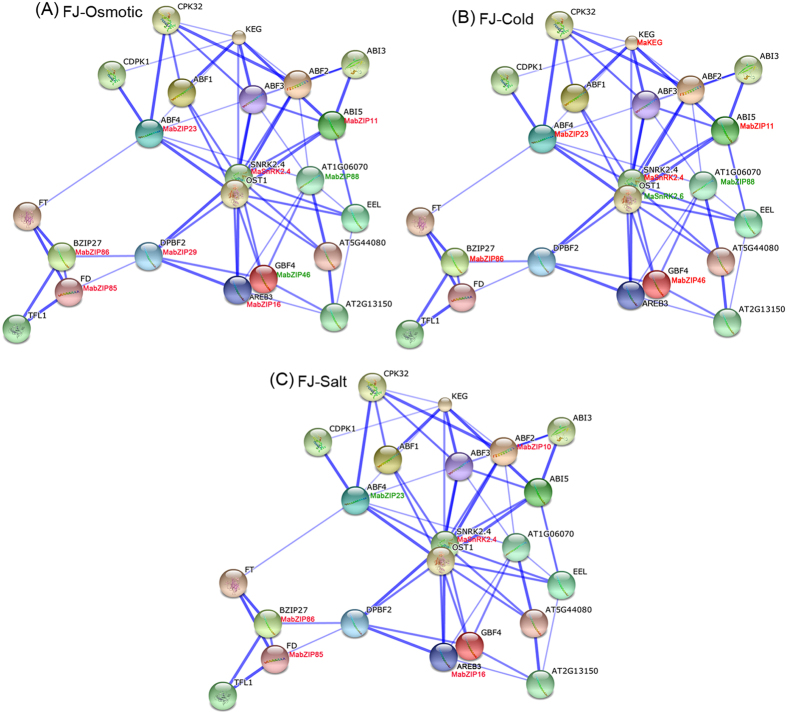
Interaction network and co-expression analyses of bZIPs under osmotic (**A**), cold (**B**), and salt (**C**) treatments in FJ and related genes in Arabidopsis. Line thickness relates to combined score. The genes marked with red show upregulation (Log2 based FPKM value > 0.5) in FJ. The genes marked with green show downregulation (Log2 based FPKM value < −0.5) in FJ.

As genes typically function in biological processes through the interaction networks, studies on gene family interaction networks are very useful for investigating potential gene function^31^. Group A bZIP genes have been documented as playing roles in abiotic stress responses in many species^16^,^17^,^18^. However, because there are no such reports in banana, we chose this group for identification of potential protein interaction and co-expression networks of banana bZIPs, aiding future studies on their biological function based on experimentally validated interactions. An Arabidopsis group A bZIP-mediated network was constructed and 22 interactive proteins (with high confidence; score > 0.9), including 12 bZIPs and 10 other interactive proteins, were identified with STRING^32^. Then, homologs of these interaction proteins in banana were identified with reciprocal BLASTP analyses and the expression profiles of these genes in BX and FJ under abiotic stress were extracted from RNA-seq data sets (Figs 8 and 9; Supplementary Tables S5 and S6). Under osmotic stress in BX, 3 gene pairs ABF2:MabZIP10-SnRK2.4:MaSnRK2.4; SnRK2.4:MaSnRK2.4-GBF4:MabZIP46; SnRK2.4:MaSnRK2.4-AREB3:MabZIP16 showed co-expression of uniform upregulation, whereas the gene pairs ABF4:MabZIP23-AT1G06074:MabZIP88 showed uniform downregulation (Fig. 8A). Under osmotic stress in FJ, 7 gene pairs showed uniform upregulation, while only one gene pairs of GBF4:MabZIP46-AT1G06074: MabZIP88 showed uniform downregulation (Fig. 9A). Under cold treatment, 3 gene pairs had downregulated co-expression in BX, whereas 4 genes pairs had upregulated co-expression in FJ (Figs 8B and 9B). Under salt stress, no gene pairs showed co-expression in BX, while 3 gene pairs had upregulated co-expression in FJ (Figs 8C and 9C). Additionally, some gene pairs had negative correlation in the gene expression, such as FD:MabZIP85-DPBF2:MabZIP29; DPBF2:MabZIP29-AREB3:MabZIP16; DPBF2:MabZIP29-SnRK2.4:MaSnRK2.4; DPBF2:MabZIP29-GBF4:MabZIP46; SnRK2.4:MaSnRK2.4-ABF4:MabZIP23 in BX under osmotic stress (Fig. 9A). Collectively, the interaction network and co-expression analyses indicated the crucial roles of Group A bZIP member-mediated network in stress signaling, and more gene pairs were uniformly up-regulated in FJ than in BX in response to the osmotic, cold and salt stresses.

## Discussion

Banana is a major popular tropical and subtropical fruit that is consumed worldwide and is also a staple crop supplied to more than 130 countries,^27^,^33^. Compared to other important crops, research on banana has evolved slowly, especially with respect to the responses to environmental stresses^27^. The *bZIP* gene family, one of the largest transcription factor families in plants, has been reported to participate in various biological processes, including the regulation of plant growth, development and ripening, as well as responses to multiple stresses. Although the *bZIP* family has been identified in many plant species, the genome-wide identification of *bZIPs* has not been reported in banana. Herein, 121 *bZIP* family genes were identified in the banana genome (*Musa acuminate*, A-genome, 2n = 22), and they were classified into 11 subfamilies according to their phylogenetic evolutionary relationship (Fig. 1), which was consistent with Arabidopsis^3^, maize^10^ and *B.distachyon*^9^. The phylogenetic analysis was also supported by conserved motif and gene structure analyses. Conserved motif analysis indicated that all the MabZIPs harbored the typical bZIP domain, and each subfamily shared similar motifs (Fig. 2). These features of the bZIP conserved motifs have also been observed in some other plants such as grapevine^6^, and cassava^34^. Gene structure analysis indicated that *MabZIPs* contain exons with numbers varying from 1 to 13, and each subfamily shared the similar exon-intron organizations (Fig. 3). It was also observed that the members of subfamilies G and D harbor more than 9 exons, whereas the other subfamily members contain fewer introns. According to a previous report on rice, the rate of intron loss is faster than the rate of intron gain after segmental duplication^35^. Thus, it can be concluded that the subfamilies G and D might contain the original genes, and the other groups were derived by gene duplication with subsequent intron loss. This gene structure feature of *MabZIP* has also been observed in other species, such as grapevine and *Brachypodium distachyon*^6^,^9^. Gene structure and conserved motif analyses indicated that *MabZIPs* in the same group had similar exon-intron organizations and conserved motifs, suggesting the *MabZIPs* in the same group had a closer relationship during the gene evolution process.

As one of the most popular fruits, fruit development and ripening process are crucial for banana fruit quality^36^. The *bZIP* family has been reported to participate in the fruit development process of many plant species such as tomato^37^, apple^38^ and watermelon^39^; however, whether *bZIPs* participate in fruit development and post-harvest ripening of banana is unclear. In this study, we found that more than 67.2% of *bZIP* genes expressed at the 5 tested development and ripening stages of BX and FJ, among which more than 16.0% genes showed high expression levels (value > 10) at each stage of the two banana varieties (Fig. 5, Supplementary Table S3). These results imply that *bZIP* genes are extensively involved in the fruit development and ripening processes of banana.

By comparing the *MabZIP* expression profiles at different stages of fruit development and ripening between BX and FJ, an interesting phenomenon was observed. BX and FJ showed similar expression patterns at 0 and 20 DAF, with no less than 80.7% *MabZIP* genes expressed at these stages, and more than 32.8% *MabZIP* genes showed high expression levels (value > 10) at these stages (Fig. 5, Supplementary Table S3). These results indicated that *MabZIP* genes had important functions at the early stage of fruit development in BX and FJ. This phenomenon has also been observed in some other plants, such as the tomato *bZIP* gene (*npr1*) was up-regulated during locular tissue and mesocarp development at 20 days post anthesis^40^.

Notably, there were more genes showed high expression levels in BX than in FJ at 20 DAF and the subsequent developmental and ripening stages, implying that the *bZIP* family had more important functions during development and ripening of BX (Fig. 5, Supplementary Table S3). The majority of edible cultivated bananas are triploid with a genome constitution of AAA, AAB and ABB, which originated from intraspecific or interspecific hybridization between wild diploid *M. acuminate* (A-genome) and *M. balbisiana* (B-genome) species^41^,^42^. The A-genome (*M. balbisiana*) has been associated with banana fruit quality, which is determined by pre-harvest development and post-harvest ripening processes^42^. BX, showing one more A-genome than FJ, is reported to have a high quality of fruit because of its high yield, long fingers, and long-term storage^36^. Collectively, these evidences indicate that the high ratio of *MabZIP* genes with high expression levels in different fruit developmental and ripening stages of BX might contribute to the fruit quality of banana.

Banana, as a tropical and subtropical plant, is extremely sensitive to water stress, which is caused by certain abiotic stress such as drought, salt or cold. Banana requires an abundant water supply in the development processes because of its permanent green canopy, shallow roots, and rapid growth rate^33^. However, few studies have reported banana abiotic stress responses. It has been reported that many bZIP transcription factors participate in the regulation of multiple abiotic stress in various species. The group A bZIP (AREB/ABFs), which can be activated by SnRKs and are involved in the PP2C-SnRK-AREB pathway, have been confirmed to be important components of the ABA signaling pathway^22^. ABFs can modulate the expression of ABA-dependent genes with the ABRE *cis*-element under ABA or abiotic stress, and play a positive role in multiple abiotic stress responses^3^,^43^. In this study, no less than 74.3% *bZIP* genes showed transcriptional changes after abiotic stress treatments, including osmotic, salt, and cold stresses in both BX and FJ (Fig. 6, Supplementary Table S4). Gene expression analysis has showed that the *bZIP* family has increased transcription levels under abiotic stresses of cold, drought and salt in some other plants such as cassava, *B.distachyon* and maize^9^,^10^,^33^. These results indicate that *bZIP* genes exhibit extensive responses to abiotic stress.

By comparing the expression patterns of *bZIPs* under abiotic stress between BX and FJ, it was clear that more genes were significantly upregulated (value > 1) in FJ than in BX under the cold and salt treatments (Fig. 6, Supplementary Table S4). Furthermore, from the interaction network and co-expression analyses, more gene pairs were uniformly up-regulated in FJ than in BX in response to the osmotic, cold and salt stresses (Figs 8 and 9; Supplementary Tables S5 and S6). The B-genome has been considered to be related to tolerances to both biotic and abiotic stresses, and *M. balbisiana* with the B-genome is demonstrated to have resistance to biotic stress caused by Xanthomonas, as well as abiotic stress caused by drought or water stress^42^,^44^,^45^. Thus, the B-genome is considered to be a target for banana breeding programs^36^,^42^. FJ, containing the B-genome, has been reported to have strong tolerances to abiotic stress^36^. Numerous evidences have confirmed that bZIPs play a positive role in plants response to abiotic stress^16^,^17^. Together, these findings suggest that the high ratio of *MabZIP* genes and gene pairs upregulated by abiotic stress in FJ could contribute to the tolerance of banana to abiotic stress.

In conclusion, we identified 121 *bZIP* genes from banana and determined their basic classification and evolutionary relationships using evolutionary, conserved protein motif and gene structure analyses, which will supply abundant information for functional characterization of *bZIP* genes. The expression profiles of *MabZIPs* in distinct tissues, stages of fruit development and ripening indicate that *MabZIP* genes have distinct functions during the fruit development and ripening of the two banana varieties, thus advancing the understanding of the molecular basis for tissue development and function. Expression patterns of *MabZIPs* responding to abiotic stress and protein interactional network analyses indicate that they are involved in abiotic stress signaling. These results will advance the understanding of the roles of bZIP transcription factors in the regulation of signal transduction pathways of banana developmental processes and responses to abiotic stress, and lay a solid foundation for further researches on banana breeding.

## Methods

### Identification and phylogenetic analyses

The whole banana (*Musa acuminata*) protein sequences were obtained from the banana genome database (http://banana-genome.cirad.fr/)^30^. The bZIP protein sequences from rice and *Arabidopsis* were acquired from the RGAP (http://rice.plantbiology.msu.edu/) and TAIR (http://www.arabidopsis.org/) databases, respectively. The HMM profiles of the bZIP domain (PF00170) (http://pfam.sanger.ac.uk/) were used as queries to search predicted bZIP proteins in the banana dataset using HMMER software (HMMER: http://hmmer.wustl.edu/)^46^. BLAST algorithm were also used to identify the predicted banana bZIPs with all bZIPs from rice and *Arabidopsis* as queries. Then, the potential banana bZIPs were further examined with PFAM (http://pfam.sanger.ac.uk/) and CDD (http://www.ncbi.nlm.nih.gov/cdd/) databases. The accession number of identified banana bZIPs could be available in Table S1. The bZIP protein sequences from banana and Arabidopsis were aligned using Clustal X2.0, and then the phylogenetic tree was constructed based on the sequence alignments using MEGA 5.0 software with bootstrap values for 1000 replicates^47^.

### Protein properties and sequence analyses

The molecular weight and isoelectric points of the MabZIPs were predicted from the ExPASy database (http://expasy.org/). MabZIP proteins motifs analyses were performed with MEME software (http://meme.nbcr.net/meme/cgi-bin/meme.cgi) and annotated by InterProScan database (http://www.ebi.ac.uk/Tools/pfa/iprscan/). The *MabZIP* gene structure analysis was conducted with GSDS (http://gsds.cbi.pku.edu.cn/). The protein-protein interactions were determined using STRING (http://string-db.org/) with the confidence score > 0.9.

### Plant materials and treatments

Two varieties of BaXi Jiao (*Musa acuminate* L. AAA group cv. Cavendish, BX) and Fen Jiao (*Musa* ABB PisangAwak, FJ) were used for this study. BX has many virtues of high production and long storage, so it is widely cultivated in China. FJ, with many virtues of good flavor and more resistance to stresses, has been widely planted in the Hainan province of China. Five-leaf stage young banana seedlings of BX and FJ were obtained from the banana tissue culture center (Danzhou, Institute of Bananas and Plantains, Chinese Academy of Tropical Agricultural Sciences), and then they were grown in soil with the greenhouse conditions of 28 °C, 70% relative humidity and 200 μmol m^−2^ s^−1^ light intensity in 16 h light/8 h dark cycle. Organs including roots, leaves at the five-leaf stage, and fruits of 80 DAF were collected from BX and FJ separately for gene expression analysis in different organs. For gene expression analysis in fruit development process, fruits of 0, 20 and 80 DAF, which represent fruits developmental stages of budding, cutting flower and harvest stages, were collected from BX and FJ, respectively. For gene expression profile analysis in post-harvest ripening process, the fruits of 8 and 14 DPH in BX and 3 and 6 DPH in FJ were sampled respectively, as it is faster for FJ reaching full yellow degree than that of BX after harvesting^36^,^48^. For abiotic stress treatments, five-leaf stage banana seedlings grown in soil were irrigated with 200 mM mannitol or 300 mM NaCl for 7 days in order to induce osmotic and salt stress, respectively. For cold treatment, banana seedlings were incubated in 4 °C for 22 h.

### Transcriptomic analysis

Total RNA of each sample were extracted with plant RNA extraction kit (TIANGEN, China), and then they were constructed into cDNA libraries, respectively. The sequencing were performed with an Illumina GAII following manufacturer’s instructions. There are two replicates for each sample. The sequencing depth was 5.34X on average. Using FASTX-toolkit, adapter sequences in the raw sequence reads were removed. After examining the sequence quality and removing low quality sequences by FastQC, clean reads were generated. Using Tophat v.2.0.10, clean reads were maped to the DH-Pahang genome (*Musa acuminate*, A-genome, 2n = 22)^30^. The transcriptome assemblies were performed by Cufflinks^49^. Gene expression levels were calculated as Reads Per Kilobase of exon model per Million mapped reads (FPKM). DEGseq was used to identify differentially expressed genes^50^.

### qRT-PCR Analysis

Changes in the expression of MabZIP genes in different organs, different stages of fruit development and ripening, and response to abiotic stresses of cold, salt and osmotic were validated by quantitative real-time polymerase chain reaction (qRT-PCR) using SYBR^®^ Premix Ex Taq™ (TaKaRa, Shiga, Japan) chemistry on a Stratagene Mx3000P (Stratagene, CA, USA) instrument. Primers with high specificity and efficiency determined by melting curve analysis and agarose gel electrophoresis were selected to perform quantification assay (Table S7), and the amplification efficiencies of primer pairs were between 0.92 and 1.17. *MaRPS2* (HQ853246) and *MaUBQ2* (HQ853254) were used as internal controls to normalize the relative expression of target genes^51^. The relative expression levels of the target genes were assessed based on 2^−ΔΔCt^ method^52^. Each sample contains three replicates.

## Additional Information

**How to cite this article**: Hu, W. *et al*. Genome-wide analyses of the bZIP family reveal their involvement in the development, ripening and abiotic stress response in banana. *Sci. Rep.* **6**, 30203; doi: 10.1038/srep30203 (2016).

## Supplementary Material

Supplementary Information
